# Ophthalmic pathology in patients with primaryneurodegenerative diseases

**DOI:** 10.1002/alz.083664

**Published:** 2025-01-09

**Authors:** Ekaterina Vladimirovna Makhnovich, Anna Nicolaevna Bogolepova, Ekaterina Аndreevna Kovalenko, Osinovskaya Nina Alekseevna, Mikhail Mikhailovich Beregov

**Affiliations:** ^1^ Federal Center of Brain and Neurotechnologies, FMBA of Russia, Moscow Russian Federation; ^2^ N.I. Pirogov Russian National Research Medical University, Ministry of Health of Russia, Moscow Russian Federation

## Abstract

**Background:**

The priority problem of modern healthcare is irreversible dementia due to the steady increase in morbidity. Among irreversible dementias, Alzheimer’s disease takes the first place. Most often, only with sufficiently pronounced cognitive disorders, the doctor can diagnose Alzheimer’s disease, although it is obvious that the neurodegenerative process begins even before the clinical manifestations of the disease. Due to the common origin of the eye and the brain, we can say that the eye is a “window to the brain”. Many scientists note the similarities between gerontophthalmological diseases and Alzheimer’s disease.

**Method:**

The study examined 60 patients who were divided into 2 groups: the first group included patients with Alzheimer’s disease, the second group included patients with vascular dementia. Each group included 9 men and 21 women, with an average age of 65.5 years. All patients underwent a comprehensive neuropsychological examination, using the short Mental Status Assessment Scale (MMSE), the Montreal Cognitive Function Assessment Scale (MoCA), the battery of tests for assessing frontal dysfunction (FAB), the clock drawing test. Also, all patients underwent ophthalmological examination of the visometry, measurement of intraocular pressure, examination of the anterior segment of the eye and fundus, and spectral optical coherence tomography.

**Result:**

In the course of the study, significant ophthalmic pathology was detected in the group with neurodegenerative changes ‐ age‐related macular degeneration was found in 16.7%, pseudoexfoliative syndrome‐20%, primary open‐angle glaucoma‐46.7%.

**Conclusion:**

This means that the risk of developing gerontophthalmological diseases increases in patients with neurodegenerative disorders. Therefore, we can assume that the retina is a mirror of neurodegenerative changes in the brain, which will allow to diagnose the disease at early stages and with the help of drug therapy to delay further development of the disease, resulting in reduced disability for patients and socio‐economic costs of society. The results obtained are promising for the early diagnosis of primary neurodegenerative diseases and indicate the need for further research on this topic.

